# Sinus Tarsi Approach for Calcaneal Fractures: A Single-Center Comparison of Early Versus Delayed Surgery by a Consultant-Led Orthopedic Team

**DOI:** 10.7759/cureus.91647

**Published:** 2025-09-05

**Authors:** Siang Yew Yeo, Fu Yuen Thong

**Affiliations:** 1 Orthopaedic Department, Queen Elizabeth II Hospital, Kota Kinabalu, MYS

**Keywords:** calcaneum fixation, calcaneum fractures, displaced intra-articular calcaneal fracture, minimally invasive sinus tarsi approach, sinus tarsi approach

## Abstract

Background

The sinus tarsi approach (STA) is a well-established technique for treating displaced intra-articular calcaneal fractures (DIACFs) due to its reduced wound complication rates compared to the extensile lateral approach. However, concerns remain regarding the efficacy and safety of STA when performed beyond the 14-day post-injury mark.

Objective

The objective of this study was to evaluate and compare the radiological outcomes and complication rates between early (<14 days) and delayed (≥14 days) STA interventions.

Methods

This retrospective study was conducted at Hospital Queen Elizabeth II, Sabah, Malaysia. A total of 42 calcanea with DIACFs treated using STA from July 2022 to July 2025 were included. Patients were divided into early (12 calcaneum) and delayed (30 calcaneum) groups based on the timing of surgery. Pre- and postoperative Böhler and Gissane angles were measured and compared. Surgical duration and postoperative complications were analysed. As this was the first series of calcaneal fractures treated using the sinus tarsi approach at our center, all eligible cases (n=42) treated between July 2022 and July 2025 were included.

Results

The mean preoperative Böhler angle was 9.5°, and it improved postoperatively to 26.6°. The mean preoperative Gissane angle was 116.9°, and it improved to 131.0° postoperatively. No statistically significant differences were found between early and delayed groups for Böhler (p = 0.426) and Gissane angles (p = 0.746). Complication rates were 16.7% for early and 3.3% for delayed groups (p = 0.192). Mean operative time did not significantly differ between the early (107.7 mins) and delayed (113.4 mins) groups (p = 0.599).

Conclusion

The STA provided consistent radiographic outcomes in both early and delayed interventions. STA remains a safe and adaptable option in our centre, though larger prospective studies with functional outcome assessments are warranted to strengthen external validity.

## Introduction

Calcaneal fractures are among the most complex fractures in orthopedics, accounting for approximately 2% of all fractures. Displaced intra-articular calcaneal fractures (DIACFs) are particularly challenging due to their anatomical intricacy and the need for precise reduction to restore hindfoot function [1].

The extensile lateral approach (ELA) has historically been considered the gold standard for surgical treatment. Despite its ability to provide excellent visualization of the fracture, it is associated with significant wound healing complications [2,3]. The sinus tarsi approach (STA) has emerged as a minimally invasive alternative with a lower complication profile, preserving soft tissues while enabling sufficient reduction of DIACFs [4,5].

However, there is ongoing debate regarding the optimal timing for STA. Some surgeons advocate restricting STA to within 14 days post-trauma, citing concerns over fracture consolidation and surgical access [6,7]. This study aims to assess whether delayed STA (≥14 days) compromises radiological correction and increases the risk of complications compared to early STA (<14 days).

## Materials and methods

Study design and setting

This was a retrospective, single-center observational study conducted at Hospital Queen Elizabeth II, a tertiary trauma referral centre in Kota Kinabalu, Sabah, Malaysia. Data were collected between July 2022 and July 2025.

Patient selection and grouping

Patients were considered eligible for inclusion if they were aged between 18 and 70 years and presented with displaced intra-articular calcaneal fractures classified as Sanders type II or III. All included patients underwent operative fixation using the sinus tarsi approach. Exclusion criteria encompassed open calcaneal fractures, Sanders type IV fractures, and fractures with an Essex-Lopresti tongue-type configuration. Extra-articular calcaneal fractures were also excluded, as were patients with significant comorbidities known to impair wound healing such as diabetic foot ulcers or peripheral vascular disease. Patients with concomitant ipsilateral lower limb fractures that required surgical intervention during the same operative session were also excluded. In addition, patients who underwent alternative surgical techniques, including the extensile lateral approach or percutaneous screw fixation, were excluded to ensure procedural uniformity across the study cohort.

Following final eligibility determination, patients were stratified into two groups based on the time interval from injury to surgical intervention. The early group comprised patients who underwent surgery within 14 days of injury (<14 days), whereas the delayed group included those who received operative treatment at or beyond 14 days and post-injury (≥14 days).

Surgical procedure

All surgeries were performed by a consultant-led orthopaedic team comprising a senior orthopaedic foot and ankle consultant, a junior foot and ankle consultant, and a subspecialty fellow in foot and ankle surgery. The senior consultant was present for all procedures. Surgeries were conducted under either spinal or general anesthesia, with patients positioned in the lateral decubitus position, ensuring the operative foot was oriented upward. A total of 5 patients presented with bilateral calcaneal fractures (10 calcanea in total). In two patients (four calcanea), the procedures were performed sequentially--first on one side in the lateral position, followed by repositioning to the contralateral lateral position for fixation of the second calcaneus. In the remaining three patients (six calcanea), both sides were operated on in a single session by two surgeons concomitantly, with the patient positioned prone. The surgical duration for each calcaneus was recorded separately.

**Figure 1:**
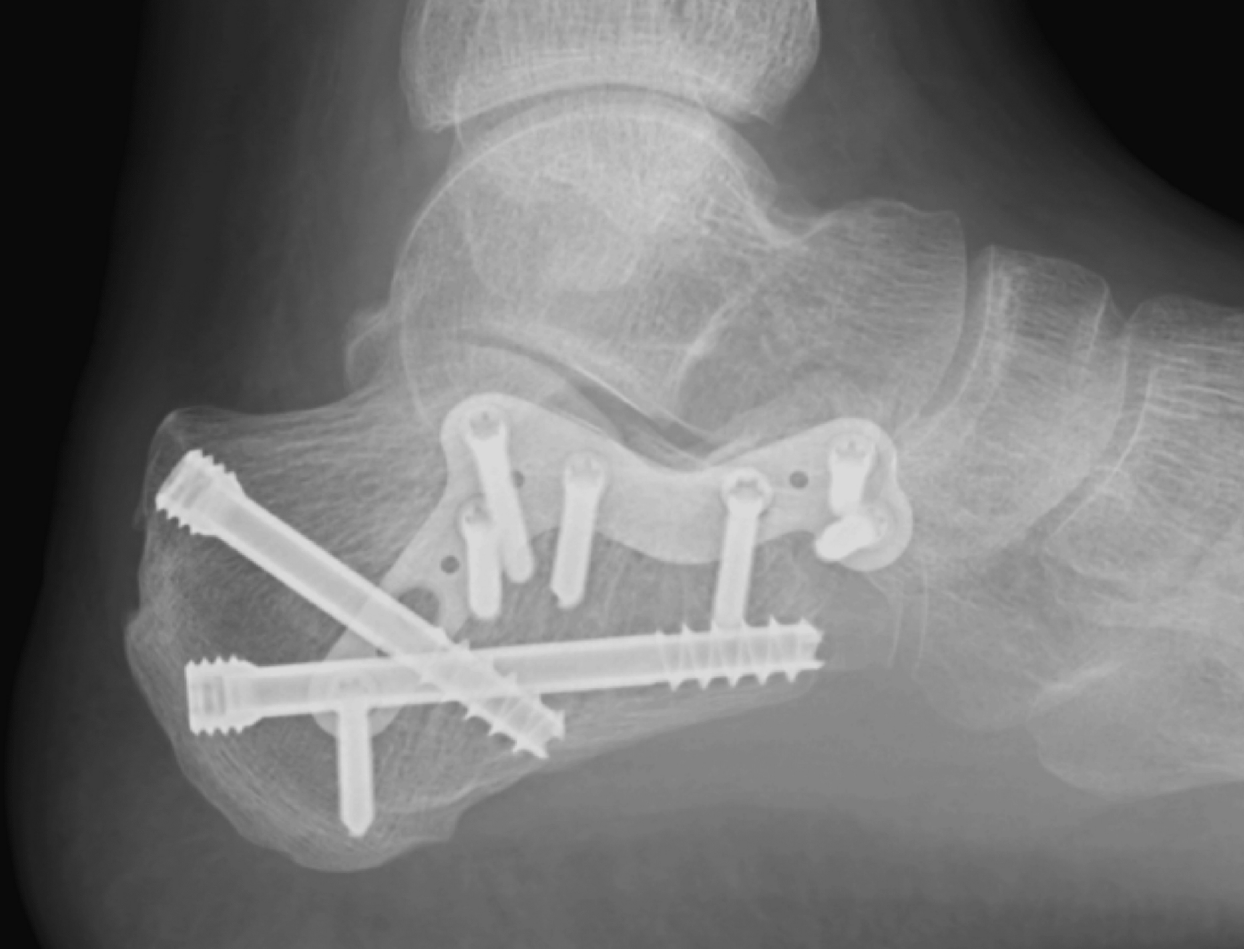
The standard implants used by the authors included a low-profile sinus tarsi plate complemented by two headless compression cannulated screws (6.5 mm or 4.5 mm)

**Figure 2:**
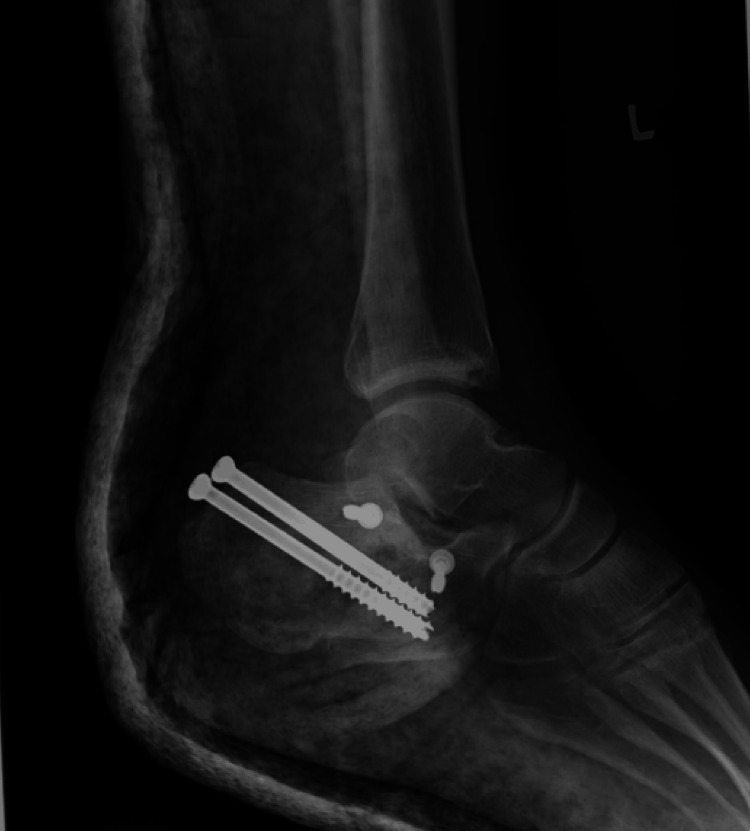
Alternative implant: all screws fixation method

**Figure 3:**
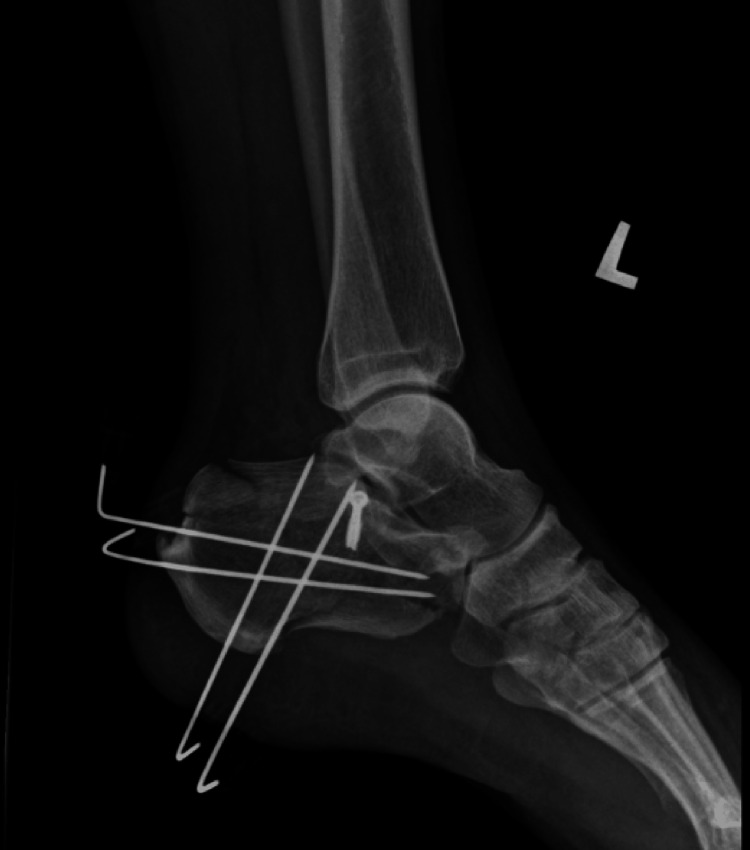
Alternative implants: screw fixation at the posterior facet and K-wires on the other fracture fragment K-wires were kept for a total of six weeks with protected weight-bearing and were subsequently removed.

A standard sinus tarsi approach was employed. A 4-6 cm longitudinal incision was made from the tip of the lateral malleolus toward the base of the fourth metatarsal. Careful dissection was performed to preserve the sural nerve and peroneal tendons. The extensor digitorum brevis muscle was elevated to expose the subtalar joint and lateral wall of the calcaneus. Fracture fragments, particularly the posterior facet, were mobilized, anatomically reduced, and temporarily stabilized with Kirschner wires. Definitive fixation was primarily achieved using a low-profile sinus tarsi plate supplemented with two large cannulated screws from the posterior calcaneal tubercle (Figure 1). For some fractures where the standard implants were not available, alternative fixation methods included three or four cannulated screws alone (three calcanea) (Figure 2). One calcaneum was fixed with a cortical screw 3.5 mm at the posterior facet, and the other fracture fragments with 2.0 mm Kirschner wires (Figure 3).

Reduction was confirmed intraoperatively using axial and lateral fluoroscopic imaging. Skin closure was performed using the Allgöwer-Donati technique. Wounds were dressed with a first layer of semi-wet gauze, followed by a dry gauze layer, and completed with a compression dressing. No suction drains were used for all cases. Wound inspection was routinely performed on postoperative day 1 for all patients. 

Follow-up 

Follow-up assessments were conducted at two weeks, six weeks, three months, six months, and one year postoperatively. Clinical and radiographic evaluations were performed to assess fracture union and monitor for postoperative complications. These included surgical site infections (superficial or deep), wound dehiscence, implant-related issues, such as loosening, breakage, or hardware prominence, post-traumatic subtalar arthritis, and nerve-related complications such as sural nerve injury. Partial weight bearing was typically initiated at the sixth week, depending on the patient’s clinical progress and radiological evidence of healing.

Outcome measures

The outcomes evaluated in this study were categorized into radiographic, procedural, and clinical domains. Radiographic assessment focused on the Böhler and Gissane angles, which were measured pre- and postoperatively using standard lateral radiographs. Measurements were independently verified by at least two orthopaedic surgeons on the angle measurement tool using the hospital's X-ray viewer system - Synapse PACS software (Fujifilm, Tokyo, Japan). This is to objectively quantify the degree of fracture reduction achieved. 

Procedural outcomes included the duration of surgery, recorded in minutes from skin incision to wound closure, to assess operative efficiency and determine whether surgical timing influenced technical complexity.

Clinical outcomes were assessed in terms of postoperative complications. These included surgical site infections (categorized as superficial or deep), implant-related complications, such as hardware prominence, loosening or breakage, the need for secondary procedures, including subtalar joint fusion, and nerve-related complications, particularly sural nerve irritation or injury. These measures were chosen to provide a comprehensive evaluation of surgical safety and efficacy across both early and delayed intervention groups.

Statistical analysis

All statistical analyses were performed using IBM SPSS Statistics for Windows (IBM Corp., Armonk, NY, USA). Continuous variables, including preoperative and postoperative Böhler and Gissane angles, as well as operative duration (in minutes), were expressed as means with standard deviations.

Comparisons between the early (<14 days) and delayed (≥14 days) surgical groups for continuous variables were conducted using independent samples t-tests, provided assumptions of normality were met. Categorical variables, including the presence or absence of postoperative complications, were analyzed using Fisher’s exact test due to the small sample size and the expected frequency of events in certain categories. Statistical significance was defined as a p-value less than 0.05.

All analyses were conducted with the intention of determining whether the timing of surgery had a statistically significant impact on radiographic correction, operative duration, or complication rates.

## Results

Demographic 

Initially, a total of 46 calcanuem were recruited for the study. However, two were excluded due to associated fractures: one with an ipsilateral talus fracture and another with associated fractures involving both upper and contralateral lower limb fractures. An additional two calcanea were excluded, as they were open fractures. Thus, the final analysis included 42 displaced intra-articular calcaneal fractures. Demographically, there were 33 males and 4 females, with a mean age of 41.1 years. There were 12 calcanea in the early group (8 males and 2 females) and 27 in the delayed group (25 males and 2 females), as shown in Table 1.

**Table 1:** Demographic data on the number of patients, mean age, and male: female ratio divided into early and delayed groups

Group	Number of Calcanea	Mean Age (years)	Male: Female
Early (<14 days)	12	39.8	8:2
Delayed (≥14 days)	30	41.7	25:2
Total	42	41.1	37

Radiographic outcomes

**Figure 4:**
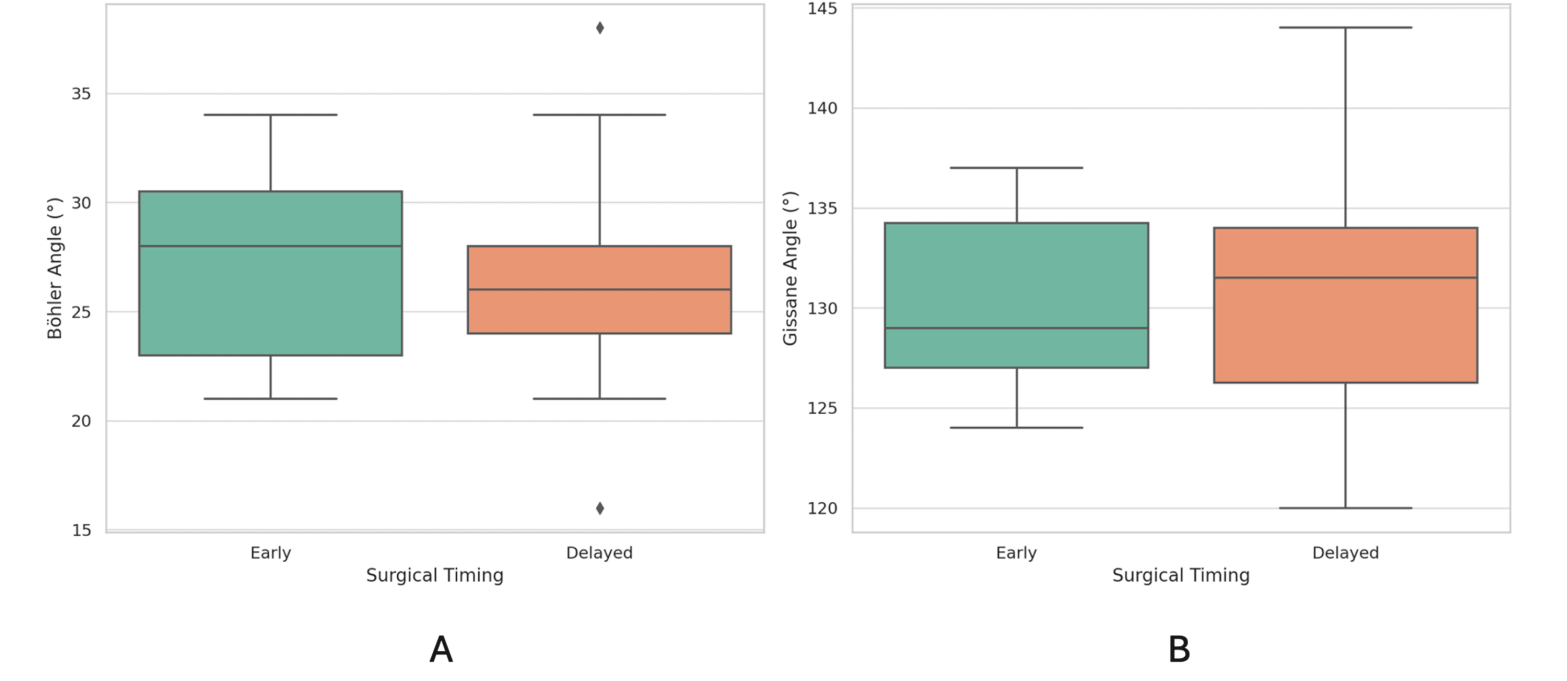
The boxplot compares postoperative Böhler and Gissane angles between the early (<14 days) and delayed (≥14 days) groups An independent t-test p-value of <0.05 shows a significant level.

Figure 4A and Figure 4B demonstrate that the postoperative Böhler and Gissane angles between the early (<14 days) and delayed (≥14 days) groups showed similar distributions with substantial overlap, indicating no significant differences in reduction quality between the two cohorts.

Table 2 further showed that the statistical analysis was consistent, as there were no significant differences in mean postoperative Böhler (p = 0.402) and Gissane angles (p = 0.682) between the two groups.

**Table 2:** Comparison of postoperative Bohler and Gissane angles (mean±standard deviation) between the early and delayed groups and their significant levels An independent t-test p-value of <0.05 shows a significant level.

Group	Preop Böhler (°)	Postop Böhler (°)	Preop Gissane (°)	Postop Gissane (°)
Early (<14 days)	10.1 ± 6.4	27.5 ± 5.4	117.3 ± 6.7	130.6 ± 6.8
Delayed (≥14 days)	9.3 ± 6.1	26.2 ± 5.9	116.8 ± 7.6	131.3 ± 6.1
P value	-	0.402	-	0.682

Duration of surgery

**Figure 5:**
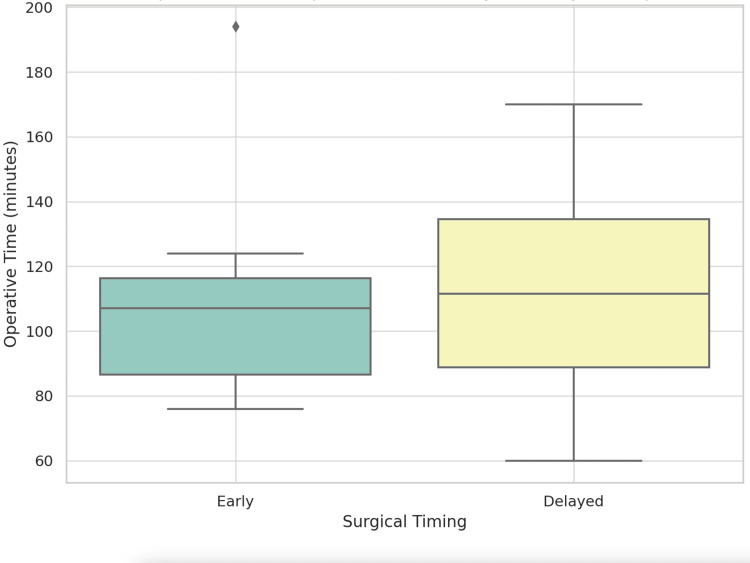
The boxplot demonstrates the operative time comparison between the early and delayed groups An independent t-test p-value of <0.05 shows a significant level.

As shown in Figure 5 and Table 3, the mean operative time was 107.7 ± 31.6 minutes in the early group (n = 12) and 113.4 ± 30.4 minutes in the delayed group (n = 30), with no statistically significant difference observed between the two (p = 0.599).

**Table 3:** Mean operative time (SD) with sample size (n) between the early and delayed groups An independent t-test p-value of <0.05 shows a significant level.

Group	Mean operative time (min)	Sample size (n)
Early	107.7 ± 31.6	12
Delayed	113.4 ± 30.4	30

Complications 

Table 4 illustrates the postoperative complications in early (<14 days) and delayed (≥14 days) sinus tarsi approach groups. For the early group, there were two complications: one with superficial surgical infection and the other with a prominent implant, which required removal. For the delayed group, there was only one complication from the STA, for which the patient required sub-talar joint fusion six months after the operation. Statistical analysis using Fisher’s exact test showed no significant difference in complication rates between groups (p = 0.192).

**Table 4:** Comparison of complications between the early and delayed groups Each complication was further elaborated. Fisher's exact test p-value of <0.05 shows a significant level.

Group	Total patients	Number of complications	Complications rate	Complications
Early	12	2	16.7%	1. Superficial surgical infection; 2. Implant prominent/irritation
Delayed	30	1	3.3%	Subtalar fusion at 6 months post-operation

## Discussion

This study confirms that the sinus tarsi approach (STA) provides satisfactory radiographic outcomes for displaced intra-articular calcaneal fractures (DIACFs), regardless of surgical timing. Improvements in Böhler and Gissane angles were consistent across both the early (<14 days) and delayed (≥14 days) groups, reinforcing the feasibility of delayed fixation when necessary [8,9]. The mean operative duration for the delayed group was 113.4 minutes, slightly longer than the early group’s 107.7 minutes, but this difference was not statistically significant (p = 0.599). Similarly, postoperative Böhler and Gissane angles showed no significant differences between groups (p = 0.402 and p = 0.682, respectively). These findings challenge the assumption that delayed surgery inherently increases technical difficulty or compromises reduction quality [10,11].

Patients in the delayed group underwent surgery later, primarily due to socioeconomic and logistical barriers. Operating theater availability was often constrained, and more critically, implant affordability and access posed significant hurdles. Standard fixation implants, including the low-profile sinus tarsi calcaneal plate and headless compression screws, were typically supplied by third-party vendors and required external funding. Financial assistance was facilitated through the hospital’s medical social welfare department, Social Security Organization (SOCSO) Malaysia, and government-linked funds such as the pensioner medical fund for retirees and the Public Service Department of Malaysia for active civil servants. These approval processes contributed substantially to the surgical delays. Additionally, some patients presented late to the hospital due to residing in remote or underserved areas with limited access to orthopaedic services, further compounding the delay in definitive surgical treatment. In certain cases, financial limitations necessitated alternative fixation strategies. Two calcaneal fractures were definitively fixed with cortical screws alone (Figure 2), and one was initially stabilized with Kirschner wires while awaiting implant approval (Figure 3). Postoperative imaging showed acceptable reductions, and the Kirschner wires were retained as definitive fixation. All three fractures were reduced and fixed using the sinus tarsi approach. These cases illustrate the adaptability of STA and demonstrate that acceptable radiological results can still be achieved with alternative implants when anatomical reduction is adequately performed [12,13].

Although the early group demonstrated a numerically higher complication rate (16.7%) compared to the delayed group (3.3%), this difference was not statistically significant (p = 0.192). The observed complications included one superficial wound infection, one case of hardware-related discomfort requiring elective implant removal, and one patient who underwent subtalar fusion due to persistent joint pain. The superficial wound infection was identified during the second postoperative week. It presented as wound gapping of less than 0.5 cm and was managed conservatively with oral antibiotics, regular wound dressing, and Steri-Strip application. The wound demonstrated progressive healing and had fully resolved by the fourth postoperative week without further complications. In one case, the sinus tarsi plate was electively removed two years postoperatively at the patient's request. The patient reported discomfort during shoe wear, which was attributed to the prominence of the implant under a relatively thin subcutaneous soft tissue envelope over the lateral hindfoot. There were no signs of infection or implant failure. Symptoms resolved following implant removal. The third complication involved a patient who developed persistent pain over the subtalar joint postoperatively. Clinical examination revealed localized tenderness at the subtalar joint, and radiographs demonstrated degenerative changes suggestive of post-traumatic subtalar osteoarthritis. The patient underwent hardware removal and subtalar joint fusion using two large cannulated screws at six months following the initial STA procedure. This case highlights the potential for post-traumatic arthropathy in patients with significant joint surface involvement or residual malalignment.

Overall, the complication profile observed in this study aligns with previously reported data supporting the low wound complication rates and the safety of the sinus tarsi approach compared to the extensile lateral approach [3,14]. Notably, no cases of sural nerve injury were recorded, underscoring the importance of careful dissection and anatomical familiarity [15]. These findings support the reliability of the STA, even in delayed presentations, as a safe and adaptable option for managing displaced intra-articular calcaneal fractures [16]. Our results align with recent studies suggesting that the quality of reduction and outcomes are not compromised by delaying STA beyond 14 days. Several prospective and retrospective studies have reported equivalent outcomes in delayed cases, supporting the long-term reliability of STA in extended time frames [9-11]. The consistency of surgical outcomes in this study may also be attributed to the consultant-led orthopaedic team, comprising a senior foot and ankle consultant, a junior consultant, and a fellowship-trained foot and ankle surgeon. This standardization likely reduced variability and enhanced procedural quality across both groups.

Limitations

Functional outcome scores, including the American Orthopaedic Foot & Ankle Society (AOFAS) score and the Manchester-Oxford Foot Questionnaire (MOXFQ), were not included in this study due to inconsistent and incomplete documentation during the follow-up period. As a result of the retrospective nature of the study and the variability in clinical recording practices, a comprehensive dataset suitable for functional outcome analysis could not be obtained. Consequently, the study focused on objective radiographic parameters, specifically Böhler and Gissane angles, as well as the incidence of postoperative complications, which served as surrogate markers of surgical success.

This study is not without limitations. First, the retrospective design inherently carries risks of selection bias and limited control over confounding variables. Second, the relatively small sample size, particularly in the early surgery group, may have limited the statistical power to detect subtle differences in outcomes between groups. Third, while radiographic angles provide objective measures of reduction quality, they do not necessarily correlate with long-term functional outcomes or patient satisfaction. Additionally, the lack of standardized implant types in a minority of cases may introduce variability, although all procedures were performed under a consistent surgical technique and team leadership.

Despite these limitations, the findings offer valuable preliminary evidence supporting the use of the sinus tarsi approach for displaced intra-articular calcaneal fractures, even when surgical intervention is delayed beyond two weeks. Future prospective studies with larger sample sizes and standardized functional outcome assessments are warranted to validate and expand upon these results.

## Conclusions

The sinus tarsi approach (STA) demonstrated consistent radiographic outcomes and a low complication profile for displaced intra-articular calcaneal fractures in our cohort, irrespective of whether surgery was performed before or after 14 days. These findings indicate that delayed fixation beyond two weeks can still be performed safely and effectively when undertaken by an experienced, consultant-led orthopaedic team. To strengthen the evidence base and assess long‑term functional outcomes, further prospective studies with larger, balanced cohorts and standardized functional measures are required.
